# Attitude towards the Practice of Female Genital Cutting among School Boys and Girls in Somali and Harari Regions, Eastern Ethiopia

**DOI:** 10.1155/2017/1567368

**Published:** 2017-03-12

**Authors:** Asresash D. Abathun, Abdi A. Gele, Johanne Sundby

**Affiliations:** ^1^Faculty of Medicine, University of Oslo, Oslo, Norway; ^2^Department of Nursing and Health Promotion, Oslo and Akershus University College, Oslo, Norway; ^3^Department of community Medicine and global Health, Institute of Health and Society, University of Oslo, Oslo, Norway

## Abstract

*Introduction*. Female genital cutting (FGC) is a harmful traditional practice that violates women's rights and threatens their health. Although much work has been done to tackle this practice in Ethiopia, the prevalence remains very high in Somali and Harari regions. This study aims to investigate the attitude towards FGC of young people (boys and girls) in Somali and Harari regions of Eastern Ethiopia.* Methods*. A cross-sectional quantitative study was carried out in Somali and Harari regions from October to December 2015. Two districts were purposely selected from the two regions, and a stratified random sampling technique was employed to select 480 subjects from the randomly selected schools.* Results*. Out of 480 questionnaires distributed, 478 (99.6%) respondents filled the questionnaires and returned them. The finding of the study reveals that 86% of study participants condemn the practice of FGC. Almost 59% of male participants from both study areas preferred to marry uncircumcised girls. Being a female and being a Muslim are significantly associated with the support toward the continuation of the FGC (*P* < 0.05).* Conclusion*. Although the study demonstrates a positive attitude towards the abandonment of FGC, there is a need to increase the knowledge about the position of Islam in FGC and to educate women about the harmful effect of FGC.

## 1. Background

Female genital cutting (FGC) is a harmful traditional practice that violates women's rights and threatens their health. The practice gained increased attention in research and policy over the last decades due to continued high prevalence despite efforts to end it [1]. More than 200 million girls and women alive today have been identified as the victims of this harmful practice [2]. The practice is most common in the western, eastern, and northeastern regions of Africa, as well as among migrants from these areas. FGC is, therefore, a global concern [2].

There is not a vivid historical justification that shows when and where people started practicing FGC. However, it is believed that the followers of both Christianity and Islam have practiced FGC for thousands of years [3]. In some literature, one might find that Ancient Egyptian had performed FGC as far back as the fifth century BC [3]. Some individuals forward their scholarly guess that FGC has its origin in the Egyptian culture. Others postulated that FGC might be begun in societies where the women were subordinate to the men [4]. Regardless of its origin, FGC has been performed by Christians, Muslims, and Animists [5]. The practice was also common in United States of America and Europe in early 19th century where cutting of genitals was used to treat psychological disorders and prevent masturbation as well as “clitoral enlargement” [3].

The FGC is predominantly practiced in 28 countries in Africa and some communities in Asia [6, 7]. The most commonly cited reasons for the practice include fear of being rejected by the community, preparing the girl for marriage, ensuring premarital virginity, and preventing marital fidelity, as well as religious support and a cultural tradition [2]. FGC has a serious physical and mental health risks for women and young girls, especially for those who have undergone extreme forms of the procedure. Immediate as well as long-term complications that girls may experience from the procedure include extreme pain, excessive bleeding, shock, infections, obstetric complications, and psychological and sexual problems [1, 8]. Other described complications also include the retention of urine, difficulties in menstruation, and impaired sexual pleasure [8].

The practice of FGC has been declining over the last three decades. Around 1 in 3 girls aged 15 to 19 today have undergone the practice versus 1 in 2 in the mid-1980s. However, not all countries have made progress and the pace of decline has been uneven. According to data from UNICEF, fast decline among girls aged 15 to 19 has occurred across countries with varying levels of FGC prevalence including Burkina Faso, Egypt, Kenya, Liberia, and Togo [9]. Moreover, a study among medical students in Egypt showed that the prevalence of FGC among female students was 14.7%, with the prevalence being higher in students from rural areas (25%) [10]. But a substantial decline in the prevalence of FGC has been reported from the countries with the already low prevalence of the practice. A study done in Yemen revealed that the practice declined from 61.9% to 56.5% among newly born girls within six-year period [10]. Girls' and women's attitudes about FGM/C also vary widely across countries. In most countries in Africa and the Middle East, the majority of girls and women think it should end [9]. A study among medical students in Egypt indicated that 58.7% of female students had a favorable attitude towards discontinuation of the practice [11]. A study done in Nigeria showed the existence of ambivalent beliefs among mothers about the practice. Although over half of the respondents (56.8%) perceived FGC as not being beneficial, 44.2% of the participants support FGC [12]. Another study in Nigeria also stated that educated mothers were found to be less likely to favor the cutting of their daughters [13].

Like many other developing countries, FGC is widely practiced in Ethiopia with the national prevalence being 74.3% [14]. The practice may be linked to maternal mortality in Ethiopia and it is considered as one of the major national problems affecting the socioeconomic development of the country [15]. Although the true magnitude of the problem and the nature of the successful interventions is not yet known in Ethiopia, there has been a drop in the prevalence rate over a relatively short time from 80 percent in 2000 to 74.3 in 2005 [14]. A study done in Amhara Region of Ethiopia showed that there is a decrease in the prevalence of FGC among women categorized into two different age groups, 45–49 and 15–24. While 77% of the women from the first group underwent FGC, it decreased to 59% in the case of the second group [16]. The study stated that education and self-empowerment were factors associated with rejecting the practice [16]. Although 95.1% of women from the age groups of 45–49 in Oromia Region were exposed to FGC, it decreased to 75.8% when it comes to the age group of 15–24 [17]. A study done in north Gondar region indicated that 79% of the women in the age group 15–49 were in favor of upholding the custom. The study also identified that 70% of urban and 80% of rural respondents from north Gondar were in favor of FGC [18]. An explorative study about the continuation of FGC in Bale Zone revealed that 26.7% of the respondents had an intention for the continuation of FGC. Religion, safeguarding virginity, tradition, and social values were the major reasons for the continuation of the practice [15, 19]. Another study in Ethiopia revealed that the uncircumcised females and those who attended secondary-level education and above were not intended to continue the practice compared to those who were not able to read and write and circumcised females [15].

National prevalence data shows that the practice is decreasing by 7% among young people under the age of 24 compared to age group 45–49 [14]. As young people make up the greatest proportion (with more than one-third of the population in sub-Saharan Africa), they represent an opportunity to accelerate economic growth and reduce poverty [20]. However, no research had been conducted on the attitude towards the practice of FGC among young people in Ethiopia, particularly in Somali and Harari's regional states. Therefore, this study aims to investigate the attitude towards the practice of FGC among young Ethiopians in Harari and Somali regions of Eastern Ethiopia.

*Theoretical Background*

*Applying Social Convention Theory to FGC*. The social norm is a social rule of behavior that all community members follow based on the expectations that others will follow. When the Social Convention Theory is applied to FGC, an initial assumption is that parents love their children and ultimately want to do what is best for them [21]. The convention theory helps us understand why mothers let their daughters go through this painful procedure, and reasons for their resistance to change [22]. The theory states that the families' decision-making process is an interdependent process where a choice made by a family is affected by others and, in turn, it affects the choice made by other families [21–23].

In communities where FGC is a criterion for marriage, nearly all girls are circumcised not only to prepare them for proper marriage but also to be socially accepted and respected. In addition, since the possibility of circumcised girls getting married is high, the families' expectation to benefit from dowry is not as such negligible. On the other hand, if a girl does not get circumstanced, she will face different problems. Apart from failing to get married, the girl will be treated as a social outcast. Under these circumstances, FGC can be seen as the best way for the parents to ensure that their daughter can have a good future. Even the girls themselves would be put under pressure to welcome this painful fate of life for they need to get accepted and be marriageable. Peer pressure may be another potential independent source for maintaining FGC. According to the Social Convention Theory, it is an equilibrium state as no family has the incentive to move away from the expectation of circumcision. A convention shift, whereby a critical mass of people abandons the practice and allows their children to marry uncircumcised women, is necessary to sustain abandonment of FGC. The challenge is, thus, to convince all families within the community and move all of them at the same time from the equilibrium state of cutting to not cutting [21–23]. The convention can only be changed if a significant number of families within a community make a collective and coordinated choice to abandon the practice so that no single girl or family is disadvantaged by the decision. According to this statement, the challenge is for families to move together. Based on the Social Convention Theory, abandonment of FGC can be achieved through organized diffusion, involving influential participants, sharing information, intensive community dialogues, and enabling community members to reflect on their own perception regarding FGC practices [21]. The Senegalese Tostan project is a good example of the success of community-based programs that seek to coordinate change within a community, which organizes public declarations of abandonment of FGC, which provided strong initial support for Social Convention Theory [23].

Therefore, to achieve FGC abandonment using Social Convention Theory, an entire community need not be persuaded; rather what is required is a motivated critical mass of people to collectively decide that they are willing to abandon the practice. This critical mass (particularly the young population) need to persuade others to commit to the idea until there are enough to act together to make a public commitment to abandon FGC.

## 2. Methods

### 2.1. Participants

The school-based cross-sectional study was conducted in Somali and Harari Regional States of Eastern Ethiopia from October to December 2015. The study areas were purposely selected from both regions. Jigjiga town is the capital of the region and it is located approximately 635 km away from Addis Ababa. It has two public and three private high schools and ten primary schools. Harar town, which is 530 km away from Addis Ababa, is the capital of Harari regional state. There are four public and two private high schools and more than 15 primary schools in the town. One secondary school and one primary school were randomly selected from schools listed above in Jigjiga and Harari town. Two-proportion formula was used, where P1 is the prevalence of FGC in Somali region which is taken as 90% and P2 is the prevalence of FGC in Harari region (80%). A 95% confidence level is desired with 80% statistical power to detect a 5% difference in the proportions. The calculation yields a sample of 480 with 20% nonresponse rate. We stratified the study subjects into gender strata and, to ensure that the number of units selected for the sample from each stratum to the number of males and females, we used proportionate stratification to select 480 (boys and girls) respondents randomly. Ten nurses (five males and five females) were selected for the data collection and two supervisors were assigned to oversee the data collection process, in addition to the principal investigator. Training on how to collect data and how to pretest it was done prior to the data collection. The questionnaire was validated by administering it to 80 students (boys and girls) in different schools which were not included in the study. In order to make respondents understand the questions well, questionnaires were translated into native languages of participants, in this case, Amharic and Somali. Inclusion criteria for the study were being male and female of the age range 16–22 and being willing to participate in the study.

### 2.2. Analysis

Once collected, the data were computerized and analyzed in SPSS Version 20. Descriptive statistics was run to calculate the mean and standard deviation for continuous variable and frequency for categorical variables. We used cross-tabulations to determine group differences. Afterwards, univariate and multivariate logistic regression was performed to determine the associations. Confidence level of 95% and a *P* value of <0.05 were considered statistically significant.

### 2.3. Ethical Clearance

Before the implementation of the research, The Norwegian National Committee for Research Ethics and Jimma University Research Ethical board approved and gave permission letter to the respective regions to allow for the implementation of the survey. Informed consent was obtained from study participants after being informed in detail about the nature and the purpose of the study. All respondents have filled the questionnaire independently with nurse assistance, and appropriate measures were taken to assure confidentiality of information both during and after data collection.

## 3. Results

### 3.1. Sociodemographic Characteristics

Four hundred and eighty questionnaires were distributed by hand and 478 were returned giving a response rate of 99.6%. The mean age of study participants was 17.4 (SD 1.34). Muslims constituted 64.6% of the subjects, followed by Orthodox (31.8%). In Harari regional state, the ethnicity of the people was mixed with Oromo ethnic group constituting 37.5%, while 26.7% and 19.2% were Amhara and Harari ethnic groups, respectively. However, 85.7% of respondents in Somali regional state were Somali ethnic. Only 2.9% of study respondents were married (Table 1).

### 3.2. Attitude towards FGC

Only 14% of study participants were in favor of the continuation of the practice. This includes 20.6% of the respondents from the Somali region and 7.9% from the Harari region. The result showed that 84.6% of the respondents from Harari region and 57.6% of respondents from Somali region knew that FGC has a health risk. In total, 66.9% of the respondents mentioned that the main reason to perform FGC is tradition, while 10.7% mentioned it is due to religious requirements, and 9.6% said it is to decrease women's sexual drive (Table 2).

### 3.3. Health Effect of FGC

Among 246 female respondents, 62.6% mentioned that FGC causes a problem during delivery. The Harari regional state respondents are more knowledgeable about the health effect of FGC than the Somali regional state respondents (Table 3). Knowledge of health effect of FGC has statistical significant association with attitude towards abandoning FGC (*x*^2^ = 21.669 *P* value < 0.005) (table is not shown).

### 3.4. Knowledge of Respondents towards FGC

Three hundred and eighty-three (80.1%) persons agreed that FGC is against the law. The interregion analysis showed that the respondents from Harari region were more likely to be aware that FGC is not supported by law compared to those in the Somali region, while in the intersex analysis more of female respondents than male respondents supported the fact that FGC is against law. The interregion and intersex analysis showed that some respondents from Somali region and more of male respondents reported that FGC is supported by their religion (see Table 4). The majority of the respondents mentioned that they will not perform FGC to their future daughter, of whom the majority were female respondents from the Harari region (see Table 4). The majority of the boys from both study areas preferred to marry the uncircumcised girl, while 22.4% of the respondents from the Somali region prefer the circumcised girl to be their wife. Among participants who answered whether their religion supports FGC or not, 92% of Muslims perceived that their religion supports FGC (*X*2 = 29.650, *P* value < 0.005) (table not shown).

### 3.5. Participant's Support towards the Continuation of FGC

The logistic regression analysis showed that gender (*P* = 0.007) and religion (*P* = 0.001) had a statistically significant association (*P* < 0.05) with favoring FGC, while region (*P* = 0.318) after adjusting age (*P* = 0.098) and educational status (*P* = 0.279) was not significantly associated with favoring FGC. Females have over two times the odds of favoring FGC compared to males (OR 2.19, CI 1.24–3.85), while being a Muslim had over seven times the odds of favoring FGC than other religions (OR 7.29, CI 2.67–19.89) (Table 5).

## 4. Discussions

This quantitative study was involved in primary and secondary school students in Somali and Harari regions of Eastern Ethiopia. The study showed that vast majority of young people (86%) in both study areas have a negative attitude towards the practice of FGC. This implies that the continuous training and awareness given at community and school level may help the young people to develop a negative attitude towards the practice. The finding of this study is in line with recent finding that, in the Somali region, 62.7% of the study participants had negative attitude towards the continuation of FGC. The study also found a significant decline in the prevalence of FGC among the young people in the Somali region of Ethiopia, which was attributed to the different awareness-raising and anti-FGC interventions implemented in the area [24]. The high proportion of the negative attitude towards the practice among young people in this study may help to apply the Social Convention Theory to increase the understanding of their parents about the harmful effect of FGC to bring them into an equilibrium state of not cutting their daughters.

This study shows that more males than females were found to have a negative attitude towards FGC. Males may not favor FGC because, in these patriarchal societies, males enjoy more freedom which may help them obtain knowledge on FGC compared to females. Contrary to this, females are affected by the culture that supports the inequality between men and women in all aspects. They do not have a freedom to learn like their male counterparts. The study shows that female respondents have over two times the odds of supporting the continuation of FGC than men (OR 2.19, CI 1.24–3.85). This result is consistent with the study in Spain which stated that FGC is maintained due to social and family pressure, transmitted from generation to generation and silenced by the victims (women themselves) [25]. Another study stated that, in communities where FGC is normative, women view the practice as an important aspect of their life [26]. In contrast to this, the study done in Somalia reveals that male respondents (96%) supported the continuation of FGC and they largely preferred to marry circumcised women [27].

This study did not find a significant difference between male and female respondents regarding reasons that motivate people to perform FGC. The findings reveal that 66.9% of male and female respondents mentioned that tradition and religion are the major factors to perform the procedure. This implies that they are living in the same environment and they are able to reflect the reasons prevalent within the society. This result is similar to the result of a prior study which stated that the commonest reason to perform FGC was tradition/culture followed by religion [28]. In areas where FGC is bounded by tradition and religion, abandonment may be difficult. However, by applying Social Convention Theory, abandonment of FGC may be achieved through an organized effort by involving religious leaders and enabling community members to reflect on their own practice regarding FGC [21]. The Senegalese Tostan project is a good example of the success of community-based programs that seek to coordinate change within a community, which organizes public declarations of abandonment of FGC [23]. Accordingly, young boys and girls are the potential groups that have the possibilities to mobilize a critical mass of people who abandon FGC by convincing their families. In addition, they will play a significant role in organizing anti-FGC clubs by involving influential participants who share information with others to create a community dialogue to seek a coordinated change within the intermarrying community to reach the abandonment of FGC.

This study states that being a Muslim had over seven times the odds of favoring FGC than other religions (OR 7.29, CI 2.67–19.89). This implies that many Muslims might believe that FGC is associated with Islam and considered it as a criterion for praying. While 97% of respondents from other religions (Christians) reported that they are not in favor of FGC. This study is supported by DHS (Ethiopia) which stated that the proportion of circumcised women is lowest among Orthodox women (54 percent) and highest among Muslim women (82 percent) [29]. Another study on the assessment of barriers to behavioral change to stop FGC among women in Somali region also supported this study by stating that religion was the major reason for the perpetuation of this practice [30].

One of the interesting findings from this study is that more than half, 58.6%, of young boys in both study areas preferred to marry the uncircumcised girl (uncut). This implies that young boys have learned in schools and from friends about sexuality and the harmful effect of FGC on sexuality. It is known, from the literature, that many women with FGC suffer trouble with desire, arousability, satisfaction, and ability to achieve orgasm in the general population [31, 32]. Thus it is not surprising that women with FGC would have problems with sexual intercourse. They thus avoid sexual contact as it exacerbates all these difficulties. This, in turn, implies that young boys do have the awareness that they would rather enjoy sexual gratification in making love with uncircumcised (uncut) girls than with the circumcised (the cut) ones. This study is supported by a study done in Spain on the impact of FGC on sexual and reproductive health of mutilated women. In a study conducted in Spain female participants have testified that though FGC affected their sexual and reproductive life, the practice is maintained due to social and family pressure [25]. Another research that agrees with the present study states the fact that there are countries where more men than women are advocating ending FGC [33]. A systematic study also supported this study by stating that the level of education of men was an indicator for men's support to ban FGC [34]. A systematic review of 15 countries also stated that many men wished to abandon this practice because of the physical and psychosexual complications to both women and men [34].

However, if a girl does not get circumcised, her family might fear that their daughter may not get married. This makes the girl accept to be circumcised so that she enjoys societal acceptance that gives her a green light to get married. Under these circumstances, FGC can be seen as the best way for the parents to ensure that their daughter can have a good future. Thus, the convention theory helps us understand why mothers let their daughter go through this painful procedure, and the theory provided reasons for mothers' resistance to change [22]. According to this theory, decision-making process is an interdependent process where a choice made by a family is affected and affects the choice made by other families [21–23]. The challenge is, thus, to convince all families within the community and move all of them at the same time from the equilibrium state of cutting to not cutting [21–23]. In this regard there is a need to work on the young population to move together; then they will have the potential to convince their families to shift their attitude of circumcision to not circumcising their daughters. Some studies viewed that men and boys play a very important role in the decision-making process, so they should be involved in measures addressing women's health [35, 36]. This will indicate that the involvement of men in the anti-FGC campaign will facilitate the abandonment of FGC. In addition, sexual and reproductive health awareness and education must focus on the health risks of FGC and it should be given at community, school, and university levels.

In general, young boys and girls are more likely to promote a new behavior if continuous training and awareness will be given on the bad effect of FGC at the school level. This study recommends that giving a reasonable voice to the young population to advance their advocacy against the practice is necessary. It is because the young population can easily convince their own families and the community to be on board in their advocacy towards the abandonment of FGC. The negative attitude towards FGC among the study participants will neutralize the bottleneck of FGC “marriageability” by adopting the Social Convention Theory in the intermarrying society to abandon FGC. The prerequisite here is that program leaders, religious leaders, and young people should have a clear mind in terms of prioritizing the actions to be taken.

The study has number of limitations. The self-reported answers might have been to some extent in social desirability bias, but there is more likelihood of the participants to give a culturally acceptable answer. Time was the other major limitation that the researcher faced in the attempt to gather the data. The researcher was forced to reschedule the program when students selected for the study missed classes. And the rescheduling had to be done in terms of the regular program of the schools. Lack of standardized questionnaire related to this specific topic might be another limitation of this study.

## 5. Conclusion

This study shows that both male and female respondents have a negative attitude towards the practice of FGC. The high proportion of students' positive attitude to abounding FGC is a hopeful sign to practice the Social Convention Theory to bring a change from the equilibrium state of cutting to not cutting. However, more work should be needed to change the attitude of females and religious leaders due to the deep-rooted existing culture. The general implication for future programs to abandon FGC needs to focus on the area where FGC and religion have a strong link. The government and the program planner must attract religious leaders' support and commitment. It will be effective if campaigns and school health education should focus on religious interpretation on female genital cutting and on the health complication of FGC. Therefore, this study highlights the importance of working on young people to offer useful insights and of incorporating the health effect of FGC in school curricula, and a collaborative work which includes the young people, the religious leaders, and the government has to be done for the total abandonment of FGC from the stated regions.

## Figures and Tables

**Table 1:** Sociodemographic characteristics of the study population in Somali and Harari regions, Eastern Ethiopia.

Characteristics	Frequency (*N* = 478)	Percentage (%)
Age		
16–18	388	81.2
19–20	80	16.7
21–22	10	2.1

Sex		
Male	232	48.7
Female	246	51.3

Educational status		
Primary	228	47.7
Secondary	250	52.3

Region		
Harari	240	50.2
Somali	238	49.8

Religion		
Muslim	309	64.6
Protestant	15	3.1
Orthodox	152	31.8
Catholic	2	.4

Ethnicity		
Somali	213	44.6
Harari	57	11.9
Oromo	96	20.1
Amhara	77	16.1
Gurage	22	4.6
Tigre	13	2.7

Marital status		
Single	462	96.7
Married	14	2.9
Divorce	2	.4

**Table 2:** Group differences in attitude towards FGC in Somali and Harari regions, Eastern Ethiopia.

Reported answers of the respondents about attitude towards FGC	Frequency (*n* = 478)	Percentage	By regions		By gender	
	Total		Harari (*n* = 240)	Somali (*n* = 238)	Male (*n* = 232)	Female (*n* = 246)
Favoring FGC	*N* = 478					
Yes	68	14.2%	19 (7.9%)	49 (20.6%)	26 (11.2%)	42 (17.1%)
No	410	85.8%	221 (92.1%)	189 (79.4%)	206 (88.8%)	204 (82.9%)

Reason for not favoring FGC	*N* = 410					
It is harmful	190	46.3%	93 (43.5%)	97 (56.4%)	94 (47.7%)	96 (50.8%)
It has health risk	280	68.3%	181 (84.6%)	99 (57.6%)	130 (60%)	150 (79.4%)
It is a culture family support it	43	10.5%	15 (7.1%)	28 (16.3%)	25 (12.7%)	18 (9.5%)
I do not know	20	4.9%	15 (7%)	5 (2.9%)	13 (6.6%)	7 (3.7%)

Reason to perform FGC	*N* = 478					
Tradition	320	66.9	181 (75.4%)	139 (58.4%)	147 (63.4%)	173 (70.3%)
Religious requirement	51	10.7	15 (6.2%)	36 (15.1%)	21 (9.1%)	30 (12.2%)
To decrease sexual drive of woman	46	9.6	7 (2.9%)	39 (16.4%)	34 (14.7%)	12 (4.9%)
To protect virginity	19	4.0	10 (4.2%)	9 (3.8%)	11 (4.7%)	8 (3.3%)
To be accepted bride to get husband	10	2.1	1 (0.4%)	9 (3.8%)	3 (1.3%)	7 (2.8%)
I do not know	32	6.7	26 (10.8%)	6 (2.5%)	16 (6.9%)	16 (6.5%)
Total	478	100%				

All percentages in reasons for not favoring FGC were above 100% due to multiple responses.

**Table 3:** Reported health effects of FGC among female respondents in Somali and Harari regions, Eastern Ethiopia.

Reported answers of the respondents about the health effect of FGC	Total respondents *N* = 246	By regions	
		Harari *N* = 133	Somali *N* = 113
Do you think FGC causes infection?	136 (55.3%)	61 (45.9%)	75 (66.4%)

Do you think FGC causes pain during sexual intercourse?	156 (63.4%)	89 (66.9%)	67 (59.3%)

Do you think FGC can cause problems during delivery?	154 (62.6%)	104 (78.2%)	50 (44.2%)

Do you think FGC can cause bleeding and painful menstruation?	110 (44.7%)	73 (54.9%)	37 (32.7%)

Do you think FGC does not cause any problem?	4 (1.6%)	3 (2.3%)	1 (0.9%)

I do not know	7 (2.8%)	4 (3.0%)	3 (2.7%)

All percentages in the health effect of FGC were above 100% due to multiple responses.

**Table 4:** Knowledge of respondents about FGC in Somali and Harari regions, Eastern Ethiopia.

Reported answers of the respondents about knowledge of FGC	Frequency (*n* = 478)	Percentage	By regions		By gender	
			Harari (*n* = 240)	Somali (*n* = 238)	Male (*n* = 232)	Female (*n* = 246)
Do you think FGC is against law?						
Yes	383	80.1	199 (82.9%)	184 (77.3%)	173 (74.6%)	210 (85.4%)
No	34	7.1	12 (5%)	22 (9.2%)	21 (9.1%)	13 (5.3%)
I do not know	61	12.8	29 (12.1%)	32 (13.4%)	38 (16.4%)	23 (9.3%)

Do you think your religion supports FGC?						
Yes	68	14.2	24 (10%)	44 (18.5%)	40 (17.2%)	28 (11.4%)
No	353	73.8	187 (77.9%)	166 (69.7%)	169 (72.8%)	184 (74.8%)
I do not know	57	11.9	29 (12.1%)	28 (11.8%)	23 (9.9%)	34 (13.8%)

Do you think marriage needs FGC?						
Yes	85	17.8	32 (13.3%)	53 (22.3%)	42 (18.1%)	43 (17.5%)
No	182	38.1	63 (26.2%)	119 (50%)	141 (60.8%)	41 (16.7%)
I do not know	211	44.1	145 (60.4%)	66 (27.7%)	49 (21.1%)	162 (65.9%)

Do you perform FGC on your daughter?						
Yes	86	18.0	24 (10%)	62 (26.1%)	43 (18.5%)	43 (17.5%)
No	392	82.0	216 (90%)	176 (73.9%)	189 (81.5%)	203 (82.5%)

If no how do you protect your daughter?		*N* = 392	*N* = 216	*N* = 176	*N* = 189	*N* = 203
By being against FGC	13	3.3%	12 (5.6%)	1 (0.6%)	7 (3.7%)	6 (3%)
By teaching the community	379	96.7%	204 (94.4%)	175 (99.4%)	182 (96.3%)	197 (97%)
Male preference for marriage	*N* = 232		*N* = 107	*N* = 125		
A circumcised girl	43	18.5	15 (14%)	28 (22.4%)		
Uncircumcised girl	136	58.6	60 (56.1%)	76 (60.8%)		
No difference	40	17.2	19 (17.8%)	21 (16.8%)		
Do not know	13	5.6	13 (12.1%)	0 (0%)		

**Table 5:** A logistic regression analysis for the association of the attitude favoring/not favoring FGC with different categorical variables in Somali and Harari regions, Eastern Ethiopia.

Variables	Not in favour of FGC	In favour of FGC	COR at 95% CI	AOR at 95% CI	*P* value
Gender					
Male	206 (88.8%)	26 (11.2%)	1	1	
Female	204 (82.9)	42 (17.1)	1.631 (0.964–2.760)	2.19 (1.24–3.85)	*P* = 0.007^*∗*^

Educational status					
Primary	191 (83.8%)	37 (16.2%)	1		
Secondary	219 (87.6%)	31 (12.4%)	1.37 (0.82–2.29)	1.37 (0.78–2.41)	*P* = 0.279

Regions					
Harari	221 (92%)	19 (8%)	1	1	
Somali	189 (79.4%)	47 (20.6%)	3.016 (1.715–5.301)^*∗*^	1.38 (0.73–2.59)	*P* = 0.318

Religion					
Muslim	246 (79.6%)	63 (20.4%)	8.400 (3.308–21.329)^*∗*^	7.29 (2.67–19.89)	*P* = 0.001^*∗*^
Other religion	164 (97%)	5 (3.0%)	1	1	

Age					
16–18	338 (87.1%)	50 (12.9%)	1	1	
19–22	72 (80.0%)	18 (20.0%)	1.690 (0.931–3.066)	1.77 (0.90–3.48)	*P* = 0.098

__^*∗*^Statistically significant at *P* < 0.05. _ _^*∗*^Reference categories are denoted by 1.0.

## References

[1] World Health organization Female genital mutilation and obstetric outcome. http://www.who.int/reproductivehealth/publications/fgm/fgm-obstetric-outcome-study/en.

[2] World Health Organization http://www.who.int/mediacentre/factsheets/fs241/en.

[3] Little C. M. (2003). Female genital circumcision: medical and cultural considerations. *Journal of Cultural Diversity*.

[4] Perron L., Senikas V., Burnett M. (2013). Female genital cutting. *Journal of Obstetrics and Gynaecology Canada*.

[5] Kaplan-Marcusan A., Torán-Monserrat P., Moreno-Navarro J., Fàbregas M. J. C., Muñoz-Ortiz L. (2009). Perception of primary health professionals about female genital mutilation: from healthcare to intercultural competence. *BMC Health Services Research*.

[6] Yirga W. S., Kassa N. A., Gebremichael M. W., Aro A. R. (2012). Female genital mutilation: prevalence, perceptions and effect on women's health in Kersa district of Ethiopia. *International Journal of Women's Health*.

[7] Dorkenoo E., Morison L., Macfarlane A. (2007). A statistical study to estimate the prevalence of female genital mutilation in England and Wales. *Summary Report*.

[8] Alsibiani S. A., Rouzi A. A. (2010). Sexual function in women with female genital mutilation. *Fertility and Sterility*.

[9] Unicef (2015). UNICEF data: monitoring the situation of children and women. *The State of the World's Children Report*.

[10] Al-Khulaidi G. A., Nakamura K., Seino K., Kizuki M. (2013). Decline of supportive attitudes among husbands toward female genital mutilation and its association to those practices in Yemen. *PLoS ONE*.

[11] Abolfotouh S. M., Ebrahim A. Z., Abolfotouh M. A. (2015). Awareness and predictors of female genital mutilation/cutting among young health advocates. *International Journal of Women's Health*.

[12] Ahanonu E. L., Victor O. (2014). Mothers' perceptions of female genital mutilation. *Health Education Research*.

[13] Alo O. A., Gbadebo B. (2011). Intergenerational attitude changes regarding female genital cutting in Nigeria. *Journal of Women's Health*.

[14] ORC Macro (2006). *Ethiopia Demographic and Health Survey 2005*.

[15] Bogale D., Markos D., Kaso M. (2015). Intention toward the continuation of female genital mutilation in Bale Zone, Ethiopia. *International Journal of Women's Health*.

[16] Rahlenbeck S. I., Mekonnen W. (2009). Growing rejection of female genital cutting among women of reproductive age in Amhara, Ethiopia. *Culture, Health and Sexuality*.

[17] Rahlenbeck S., Mekonnen W., Melkamu Y. (2010). Female genital cutting starts to decline among women in Oromia, Ethiopia. *Reproductive BioMedicine Online*.

[18] Alemu S. D., Moland K. M., Talle A. (2007). FGM and Harmful traditional practice. *Evaluation Report*.

[19] Bogale D., Markos D., Kaso M. (2014). Prevalence of female genital mutilation and its effect on women's health in Bale zone, Ethiopia: a cross-sectional study. *BMC Public Health*.

[20] Hervish A., Clifton D. (2012). *Status Report: Adolescents and Young People in Sub-Saharan Africa. Opportunities and Challenges*.

[21] Mackie G., LeJeune J. (2009). *Social Dynamics of Abandonment of Harmful Practices: A New Look at the Theory*.

[22] Innocenti Insight (2010). *The Dynamics of Social Change towards the Abandonment of Female Genital Mutilation/Cutting in Five African Countries*.

[23] Shell-Duncan B., Herniund Y. (2006). Are there “stages of change” in the practice of female genital cutting?: Qualitative research findings from Senegal and The Gambia. *African Journal of Reproductive Health*.

[24] Gebremariam K., Assefa D., Weldegebreal F. (2016). Prevalence and associated factors of female genital cutting among young adult females in Jigjiga district, eastern Ethiopia: a cross-sectional mixed study. *International Journal of Women's Health*.

[25] Ballesteros Meseguer C., Almansa Martínez P., Pastor Bravo M. D. M., Jiménez Ruiz I. (2014). The voice of women subjected to female genital mutilation in the Region of Murcia (Spain). *Gaceta Sanitaria*.

[26] Isman E., Ekéus C., Berggren V. (2013). Perceptions and experiences of female genital mutilation after immigration to Sweden: an explorative study. *Sexual and Reproductive Healthcare*.

[27] Gele A. A., Bø B. P., Sundby J. (2013). Have we made progress in Somalia after 30 years of interventions? Attitudes toward female circumcision among people in the Hargeisa district. *BMC Research Notes*.

[28] Garba I. D., Muhammed Z., Abubakar I. S., Yakasai I. A. (2012). Prevalence of female genital mutilation among female infants in Kano, Northern Nigeria. *Archives of Gynecology and Obstetrics*.

[29] Central Statistical Agency (CSA) (2016). *Ethiopia Demographic and Health Survey 2016: Key Indicators Report*.

[30] Mohamed M. (2015). *Assessment of Barriers of Behavioral Change to Stop FGM Practice among Women of Kebribeyah District, Somali Regional State, Eastern Ethiopia*.

[31] Elnashar A. M., EL-Dien Ibrahim M., Eldesoky M. M., Aly O. M., El-Sayd Mohamed Hassan M. (2007). Sexual abuse experienced by married Egyptian women. *International Journal of Gynecology and Obstetrics*.

[32] Ashimi A., Aliyu L., Shittu M., Amole T. (2014). A multicentre study on knowledge and attitude of nurses in northern Nigeria concerning female genital mutilation. *European Journal of Contraception and Reproductive Health Care*.

[33] Varol N., Fraser I. S., Ng C. H. M., Jaldesa G., Hall J. (2014). Female genital mutilation/cutting—towards abandonment of a harmful cultural practice. *Australian and New Zealand Journal of Obstetrics and Gynaecology*.

[34] Varol N., Turkmani S., Black K., Hall J., Dawson A. (2015). The role of men in abandonment of female genital mutilation: a systematic review. *BMC Public Health*.

[35] DeJong J., Jawad R., Mortagy I., Shepard B. (2005). The sexual and reproductive health of young people in the Arab countries and Iran. *Reproductive Health Matters*.

[36] Mohammadi G., Amiraliakbari S., Ramezankhani A., Majd H. A. (2011). Poor reproductive health among a group of socially damaged Middle Eastern women: a cross-sectional study. *International Journal of Women's Health*.

